# Effect of Fortified Formula on Growth and Nutritional Status in Young Children: A Systematic Review and Meta-Analysis

**DOI:** 10.3390/nu14235060

**Published:** 2022-11-28

**Authors:** Paige G. Brooker, Megan A. Rebuli, Gemma Williams, Beverly S. Muhlhausler

**Affiliations:** Health and Biosecurity, Commonwealth Scientific and Industrial Research Organisation (CSIRO), Adelaide, BC 5000, Australia

**Keywords:** young children, young child formula, follow-on formula, fortified milk, growth, infant nutrition

## Abstract

Previous reviews of the effect of young child formulas on health outcomes in infants and toddlers have been inconclusive. In this study, we undertook a contemporary synthesis of studies investigating the effects of consuming fortified milk beverages (compared to cow’s milk or unfortified comparator formula) on growth and/or nutritional status in children 1–3 years of age. Five electronic databases were searched (PubMed, Web of Science, Scopus, ProQuest, and Cochrane Library) for randomised controlled trials comparing fortified milk against control milk in young children (9–48 months), published between January 1990 and June 2022. Outcomes were growth, body composition, biochemical markers, and/or nutritional status. Mean differences (MD) were pooled using random-effects meta-analysis where there were ≥3 studies. The risk of bias was assessed using the Cochrane Risk of Bias 2.0 tool. Nineteen articles (12 studies; *n* = 4795) met the inclusion criteria. Heterogeneity was substantial, likely attributable to considerable variation in study characteristics. Fortified milk was associated with increased weight gain (MD = 0.14 kg [95% CI 0.06, 021], *p* = 0.0003) compared with control milk. Subgroup analyses demonstrated increases in weight in lower-income countries, and in studies with intervention periods > 6 months. There were no effects of fortified milks on other anthropometric measures. Haemoglobin (MD = 3.76 g/L [95% CI 0.17, 7.34], *p* = 0.04) and ferritin (MD = 0.01 nmol/L [95% CI 0.00, 0.02], *p* = 0.02) concentrations were increased in infants consuming fortified milks. Fortified milk beverages appear to offer a safe and acceptable source of complementary nutrition as a short-term strategy for addressing nutritional deficits and may modestly promote weight gain in vulnerable populations when provided for periods > 6 months. This study was prospectively registered with PROSPERO (CRD42022339920) and funded by the Infant Nutrition Council.

## 1. Introduction

Nutrition during the first 1000 days, defined as the period from conception until a child’s second birthday, is widely recognised as having a major influence on health outcomes through the life course. Consequently, international health agencies, including the World Health Organisation (WHO), have established a series of guidelines aimed at ensuring that infants and young children receive optimal nutrition during this critical window [1]. Since breastmilk is universally acknowledged as the optimal source of nutrition for young infants, exclusive breastfeeding is recommended for infants from birth to six months, with continued breastfeeding until 2 years of age or beyond [2,3]. Food-based dietary guidelines recommend the introduction of solids at around 6 months of age (and not before 4 months) with particular attention given to ensuring that infants are exposed to a variety of age-appropriate nutrient-dense and iron-rich food during weaning, to ensure nutritional needs continue to be met [3].

By 12 months of age, it is recommended that young children should be consuming a variety of nutritious solid family foods which are compliant with dietary guidelines and provide the appropriate balance of nutrients [2,3,4]. However, it is well-established that compliance with dietary guidelines is universally low across a wide range of both higher- and lower-income countries, which places infants at risk of missing out on essential macro- and micro-nutrients during this critical developmental period [5,6]. In support of this, large observation studies conducted in high-income countries including the US, UK, and Australia reported that vegetable intakes in toddlers aged 12–24 months failed to meet dietary recommendations, whilst intakes of sugar-sweetened beverages and sodium exceeded them [7,8,9,10]. In separate study populations, approximately 20% of children failed to meet recommended dietary intakes of iron [6,11,12], and around 80% for vitamin D [8]. Iron deficiency anaemia is a global health problem, particularly among young children in Asia and Africa. Findings from a recent meta-analysis suggest that being from an anaemic mother, having low birth weight, and not drinking fortified milk were factors associated with iron deficiency in children under the age of five [13]. Along with these micronutrient deficiencies are concerns related to overnutrition, with the global prevalence of overweight in children under five years of age at 5.7% (38.9 million) in 2020, up from 38.2 million in 2019 [14,15]. The situation is even more challenging in lower-income countries, where undernutrition, in conjunction with micronutrient deficiency, remains alarmingly prevalent. In 2019, over 45% of cases of child mortality worldwide were linked to inadequate infant nutrition [16], and around a third of children under five were affected by stunting or wasting as a result of malnutrition in infancy [2,14].

To overcome these nutritional challenges in infants and toddlers, a variety of fortified foods and beverages have been developed that are specifically aimed at overcoming commonly identified nutritional deficits [5,17]. One such category includes a range of fortified milk-based beverages, collectively referred to as “young child formula”, “growing up milk” or “toddler milk”, which are specially targeted towards providing toddlers (1–3 years of age) with adequate intakes of essential nutrients that may be difficult to obtain from dietary intake alone. These “young child formulas” are generally positioned as a substitute for cow’s or human milk, or as a supplementary dietary component where nutritional intake is inadequate [18]. A wide variety of these “young child formulas” are now available, which may be animal or plant-based (predominantly from cow or soy), and which are typically fortified with a range of nutrients, including vitamins, minerals, omega-3 and omega-6 fatty acids, protein, and fibre [2,3,19]. There is general agreement that young child formulas may be beneficial in situations where infants are unlikely to achieve adequate nutrition through dietary intake alone, such as where access to a safe food supply is compromised or in young children with complex or high nutritional requirements. However, most infant feeding guidelines in high-income countries have suggested that there are limited benefits of consuming these products in otherwise healthy infants consuming a typical range of family foods. To date, however, no adverse effects of consuming these milks have been reported, raising the question of whether including these milks as part of a healthy balanced diet may have benefits for specific subgroups of otherwise healthy infants, such as those at risk of inadequate dietary intake or mild nutritional deficiencies [17,19,20]. 

While previous reviews have attempted to synthesise the existing evidence relating to the effects of fortified milk or foods on health outcomes, the generalisability and transferability of their findings are limited by their inclusion of studies conducted with infants from six months of age (i.e., outside the target age group), focus on infants with specific health conditions (e.g., iron deficiency anaemia) and/or inconsistent comparator groups [17,19,20]. Therefore, the aim of this study was to undertake a comprehensive systematic review and meta-analysis of all randomised controlled trials conducted in apparently healthy infants 9–48 months of age that investigated the effect of consuming a young child formula (in comparison to cow’s milk or an unfortified comparator child formula) on infant growth and nutritional status. 

## 2. Materials and Methods

The systematic review and meta-analyses were conducted and reported in accordance with the guidelines of the Preferred Reporting Items for Systematic Reviews and Meta-analysis (PRISMA) statement [21] (see Supplementary Table S1) and was prospectively registered with PROSPERO (CRD42022339920).

### 2.1. Information Sources and Search Strategy

A literature search was conducted in June 2022 across five databases: PubMed, Web of Science (core collection), Scopus, ProQuest, and Cochrane Library. In addition, searches of Google Scholar (retrieving the first 250 results), and of the Clinical Trials Registries database were conducted to ensure that relevant studies were captured. The search strategy was developed by the authors in conjunction with an expert librarian using the PICOS model (Patient, Intervention, Comparison, Outcome, and Study Type) [22]. A combination of MeSH (medical subject headings) terms and free-text keywords were used to search for relevant interventions (‘e.g., growing-up milk’, ‘follow-on formula’, ‘fortified milk’) and the outcomes of interest (e.g., ‘growth and development’ ‘body size’). The detailed search strategy is available in Supplementary Table S2. The search included original literature published from 1 January 1990 through 16 June 2022. A supplementary search for relevant studies was conducted by hand-searching the reference list of the screened manuscripts. 

### 2.2. Study Eligibility Criteria

Records were included if they met the following criteria:

1. Population was apparently-healthy children aged between 9 and 48 months at recruitment;
2. intervention group received fortified milk or formula;
3. comparison group received non-fortified milk or formula;
4. outcomes included growth parameters and/or biochemical markers; and
5. studies were randomised controlled trials (RCT).

Records were excluded if they were published before 1990, written in a non-English language, or the intervention period was less than three months. These criteria are described in more detail in Table 1. 

### 2.3. Study Selection and Data Extraction

Citations and abstracts of all retrieved records were imported to EndNote (X9) [23]. Duplicate records were identified and removed, and the remaining citations were imported to Covidence [24]. Records were assessed for eligibility with a hierarchical approach; initially screened based on their title and abstract and confirmed by full-text review. Study selection was performed by two reviewers (PB and MR) independently. Any conflicts in the selection process were resolved by discussion until a consensus was reached.

A standardised data extraction template was created in Microsoft Excel^®^ (Version 2022), and used to collect the following (where reported/applicable): (i) Publication Details: first author’s family name and year of publication, and research funding source; (ii) Study Characteristics: primary objective, study duration, randomisation procedures, blinding, treatment allocation concealment, and geographical setting; (iii) Participant Characteristics: sample size, attrition and reasons, sex distribution, age range recruited and mean age, inclusion and exclusion criteria, co-conditions, and baseline anthropometric measurements; (iv) Intervention Characteristics: details regarding intervention and control treatments (amount and type of milk prescribed and consumed), and intervention adherence; (v) Outcome Measures: methods used to assess outcomes, and outcome results; and (vi) Study Conclusions: main conclusions as reported by authors. 

Data from each study were extracted by one of two investigators (MR or GW) and reviewed for errors and inconsistencies by a third investigator (PB). Uncertainties were resolved by discussion between the investigators, as required. For each relevant outcome, the mean and standard deviation (SD) at baseline, end of the intervention, and change (where reported) were extracted for the intervention/s and control arms. When multiple time points were reported, only the end of the intervention point was used. For single studies with multiple published articles reporting different outcome measures, data were extracted separately. Where the same outcome measure was reported across multiple publications for the same study, data were only included for analysis once, in which case, the primary study was given priority over reports with secondary analyses. Studies that included multiple intervention conditions were included, if relevant (such as formulas with different concentrations of micronutrients). Data from comparator groups that did not fit the eligibility criteria (such as red meat), were not extracted and those data were not included in study analyses.

### 2.4. Data Synthesis

All outcomes were converted to the same unit of measure and expressed in SI Units whenever possible. Where applicable, established conversion factors were used to convert values from Conventional Units to SI Units. For each outcome, the change (Mean (Standard Deviation)) from baseline to the intervention’s primary endpoint was entered into Review Manager (RevMan; version 5.4.1, Cochrane, Copenhagen, Denmark) [25] for all groups. Where required, SD was calculated from reported standard errors or Confidence Intervals (CIs), or imputed as the average SD of similar studies, as proposed by the Cochrane Handbook for Systematic Reviews of Interventions [26]. When data were reported in different units (such as median and percentiles), they were transformed accordingly for purposes of uniformity [27]. SDs were imputed for four studies [28,29,30,31].

In cases where only baseline and end data were reported, the mean change was calculated by subtracting the baseline from the end value. Where the change in SD was not reported, it was imputed by using the change from baseline SDs for the same outcome measure from other studies in the review. The correlation coefficient (corr) was calculated according to the Cochrane Handbook for Systematic Reviews of Interventions [26]: *corr* = (SD_baseline_^2^ + SD_end_^2^ − SD_change_^2^)/(2 × SD_baseline_ × SD_end_). Correlation coefficients for growth outcomes were calculated from the study published by Chatchatee et al. [32]. Correlation coefficients of 0.72 and 0.81 were used for body weight and length, respectively. SDs were imputed for three studies [28,29,30]. A correlation coefficient of 0.56 was used for body mass index (BMI) and used to impute SDs for one study [30]. The average SD of similar studies was calculated, and used to impute the SDs for two studies ([28], BMI; and [30], weight- and length-for-age and weight-for-length). Correlation coefficients for biochemical measures were calculated from the works of Akkermans et al. [33] (corr = 0.53 for Haemoglobin (Hb); and corr = −0.39 for serum ferritin) and Virtanen et al. [34] (corr = 0.79 for serum transferrin; and corr = 0.70 for transferrin receptor). SDs were imputed for five studies for Hb [29,31,35,36,37], three studies for serum ferritin and serum transferrin [31,35,36], and one study for soluble transferrin receptors [37].

### 2.5. Study Quality Appraisal

All studies were assessed for their methodological quality using the Cochrane Risk of Bias Tool 2.0 for intervention studies [38] by two independent reviewers (MR and GW). Disagreements were resolved by consensus between the two reviewers (MR and GW) and, where needed, discussion with a third reviewer (PB). Judgement of the study quality were classified as ‘low’ or ‘high’ risk of bias or expressed as ‘some concerns’.

### 2.6. Statistical Analysis

Meta-analysis was performed using RevMan (version 5.4.1, Cochrane, Copenhagen, Denmark) when ≥3 studies reported the relevant data on a single outcome. When trials reported multiple comparisons relevant for inclusion in the meta-analysis, and one group was used more than once as a comparison group, the sample size of the specific group was divided by the number of times the group was used as a comparison to avoid data duplication and provide appropriate weighting for the results [26]. Heterogeneity was assessed by the I^2^ statistic. A random-effects model was chosen in preference to a fixed-effect model to address the presence of substantial heterogeneity (I^2^ values > 50%) [39]. Forest plots of weighted mean differences and 95% confidence intervals were generated using the inverse variance statistical method. A significance level of *p* < 0.05 was set throughout the meta-analysis.

#### 2.6.1. Sensitivity Analyses

Multiple sensitivity analyses were performed to assess the robustness of the pooled result. It has been suggested that the heterogeneity statistic I^2^ can be biased in small meta-analyses [40], therefore, funnel plots were also assessed graphically for publication bias. Sensitivity analyses considered: study weighting (i.e., the impact of each study on the overall result by leaving out one study at a time), study quality (trials with a high risk of bias), and trials with imputed data as factors that could have affected the overall (pooled) result. 

#### 2.6.2. Subgroup Analyses

Subgroup analyses were conducted based on the economic status of the country where the study was conducted (higher-versus lower-income countries), and for intervention length (≤6 months versus >6 months).

## 3. Results

**Figure 1:**
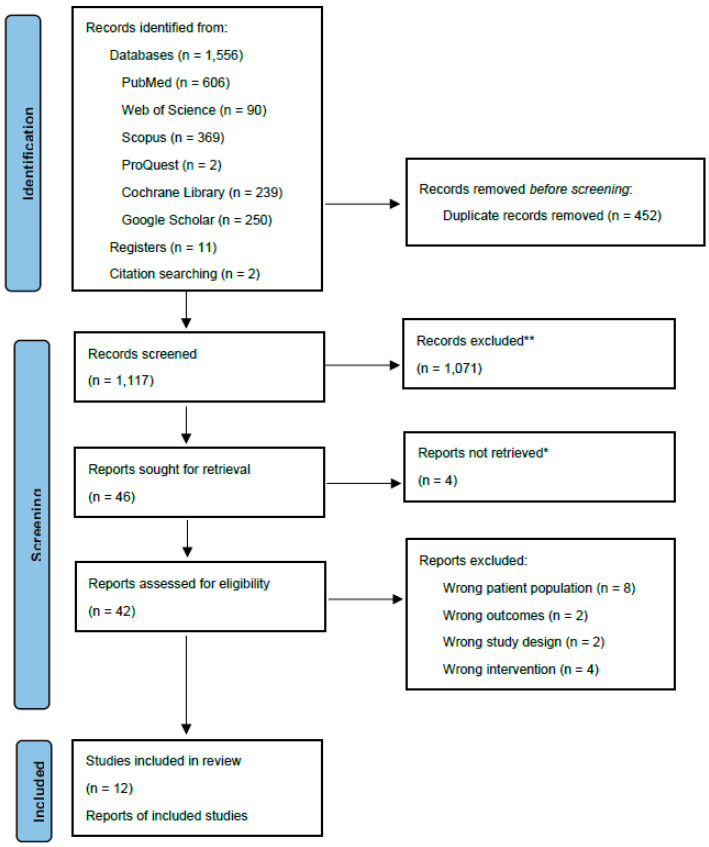
PRISMA flow chart for study selection. * Reports not retrieved refer to abstract-only records. ** Records excluded.

The literature search resulted in a total of 1569 abstracts. After the removal of duplicates (*n* = 452), a total of 1117 abstracts were initially screened by title and abstract. Forty-six abstracts were eligible for full-text review. A total of 12 studies reported across 19 publications met the eligibility criteria and were included in the review (Figure 1). 

### 3.1. Study Characteristics

The characteristics of the twelve included studies are presented in Table 2. All studies were RCTs that used a parallel-arm design. Three studies were conducted in Latin America [37,41,42], three in Asia [28,35,36], three in Europe [29,33,34], two in the Pacific region [30,43], and one study was a multi-centre trial across several countries in Asia and Europe [32]. 

The twelve studies included a total of 4795 participants. All studies included mixed-sex populations; in those of which that reported the distribution of males and females, the sex ratio was similar (range 39% to 57% males). At enrolment, the age of participants ranged from nine months up to 48 months. Most studies enrolled ‘apparently healthy’ young children, however, studies varied in their classification of ‘healthy’, or did not elaborate on this criterion. Some studies only enrolled children if they were within one standard deviation of their respective growth charts (e.g., weight-for-age z-score) according to WHO growth standards, whereas other studies enrolled children who were ‘not experiencing severe malnutrition’. Common exclusionary criteria included intolerance or allergy to cow’s milk, low birth weight or born pre-term, anaemia, and other chronic/serious illnesses including cardiovascular, gastrointestinal, renal, neurological, or metabolic. Studies varied in their classification of anaemia, one study excluded children with baseline haemoglobin concentrations < 90 g/L, whereas other studies used cut-offs of 100 g/L and <105 g/L, and some studies did not screen potential participants for anaemia, and did not apply this as a criterion for exclusion.

The duration of the interventions ranged between 20 weeks (~5 months) and 12 months. However, one study with a 12-month intervention period reported a change in the relevant outcomes at four months only [28].

The intervention characteristics and outcome measures collected across the 19 articles are presented in Table 3. Intervention milks were either those specifically marketed as ‘young child formula’ or ‘growing up milk’, or milks fortified with additional micronutrients/compounds. Common fortificants included iron, vitamins C and D, zinc, iodine, prebiotics, probiotics, and essential fatty acids. One study [30] prescribed ‘lite’ growing-up milk to intervention participants; a formula with reduced protein compared with cow’s milk. Most control groups received standard cow’s milk, either in powder or fluid form. Two studies provided participants in the control group with a non-fortified milk formula, which contained the ‘regular amount’ of micronutrients [42,43]. The dose of milk prescribed varied across the studies, ranging from >150 mL/day to 750 mL/day, and two studies prescribed ad libitum milk intake [29,34]. Two-thirds of studies (*n* = 8 of 12) included adherence check/s during the intervention to assess children’s milk consumption. 

The most common growth outcomes reported across the articles were body weight and length. Iron was the most investigated biochemical marker, followed by zinc and vitamin D, and one study measured urinary iodine. Dietary intake was reported across nine articles; energy and iron intakes were most frequently reported, followed by macronutrients and zinc, and other micronutrients (Table 3).

Regarding the risk of bias, thirteen articles were considered to have ‘some concerns’, four were deemed ‘low risk’, and two ‘high risk’ (Table 2 and Supplementary Figure S1).

Some articles were not included in the meta-analysis due to the fact that either (1) articles presented duplicate data from another article which was already included in the meta-analysis (i.e., secondary analyses of original research); or (2) data were not available in the required format and could not be imputed. Details are provided in Supplementary Table S3. Where original research study data could not be included in the meta-analysis, results are described. 

### 3.2. Effect of Formula on Infant Growth and Body Composition

Body weight and length were the most reported metrics of growth. BMI and body composition, including fat mass and fat-free mass and body fat percentage, and measurements (circumference, cm) of the head, waist, and mid-upper arm were less frequently reported. 

#### 3.2.1. Anthropometric Outcomes

**Figure 2:**
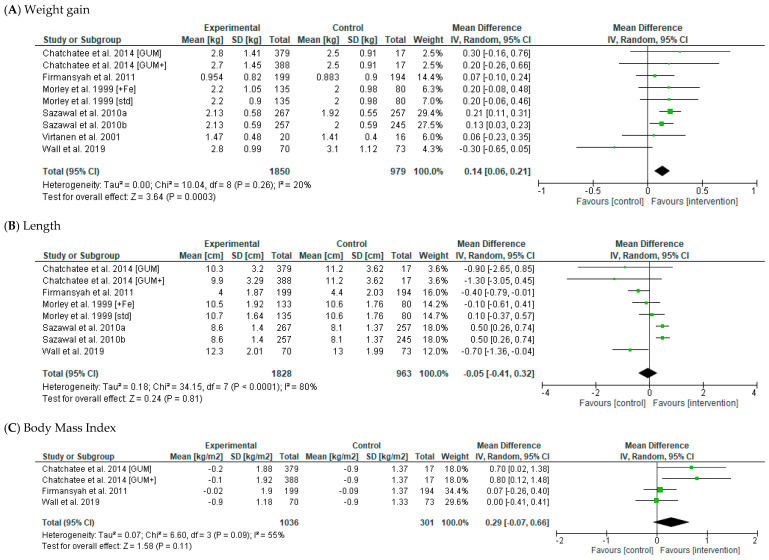

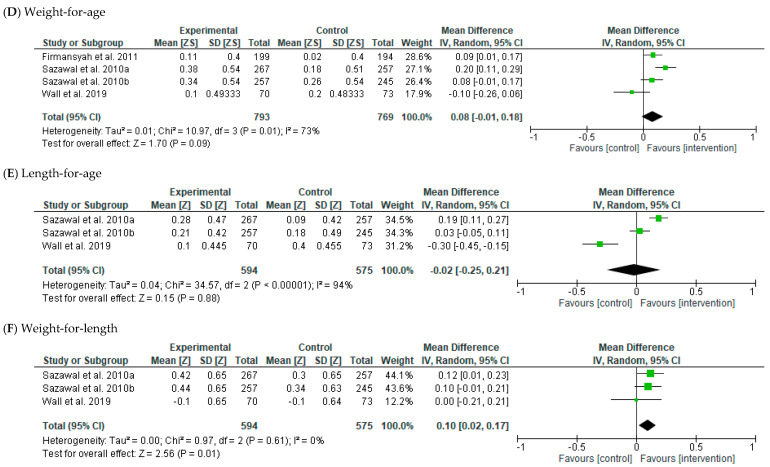
Forest plot displaying the effect of fortified milk (intervention) compared with control milk on the mean difference in (**A**) weight gain (kg) [28,29,30,32,34,35,43]; (**B**) length (cm) [28,29,30,32,34,35,36]; (**C**) body mass index (kg·m^2^) [28,30,32]; (**D**) weight-for-age (z-score) [28,30,35,36]; (**E**) length-for-age (z-score) [30,35,36]; (**F**) weight-for-length (z-score) [30,35,36]. Note: The study-specific mean differences and 95% confidence intervals are represented by the green squares and horizontal lines, respectively. The size of the square represents the weight given to each study in the meta-analysis. The shaded diamond represents the pooled mean difference, and the width of the diamond represents the pooled 95% confidence interval.

The effect of the intervention on body weight (kg) was reported as an outcome in nine articles, seven of which were included in the meta-analysis [28,29,30,32,34,35,36]. The results of this meta-analysis indicated that participants in the ‘fortified milk’ group had a greater mean weight gain following the intervention period compared to controls (Mean Difference (MD) 0.14 kg [95% Confidence Interval (CI) 0.06, 0.21], *p* = 0.0003, random effects model; 7 studies, *n* = 2829 participants) (Figure 2 and Table 4). There was a low heterogeneity between studies (I^2^ = 20%, *p* = 0.26). There was only one article [30] in the meta-analysis which favoured the control condition (albeit not significantly); when this study was removed, heterogeneity was null (I^2^ = 0%, *p* = 0.84), and there was a similar overall difference in mean weight gain between the fortified milks and control groups (MD 0.16 kg [95% CI 0.10, 0.22], *p* < 0.0001; 6 studies, *n* = 2686). In subgroup analyses, the difference in weight gain between the control and fortified milk groups was significant for studies > 6 months in duration (MD 0.15 kg [95% CI 0.06, 0.25], *p* < 0.0001; I^2^ = 31%, 5 studies, *n* = 2400) but was not seen in studies ≤ 6 months (data not shown). Similarly, the difference in body weight between control and fortified milk groups was significant for studies conducted in lower-income countries (MD 0.16 kg [95% CI 0.08, 0.23], *p* = 0.001), but not those conducted in higher-income countries (Table 4).

Length/height (cm) was reported as an outcome in seven articles, six of which were included in the meta-analysis [28,29,30,32,35,36]. There was no difference in linear growth during the intervention period between the fortified milk and control groups (MD −0.05 cm [95% CI −0.41, 0.32], random effects model, *p* = 0.81; 6 studies, *n* = 2791, Figure 2). There was high heterogeneity between studies (I^2^ = 80%, *p* < 0.0001 Figure 2), and this remained following the removal of individual studies from the analysis. Changes in linear growth were also not significant in subgroup analyses for intervention length and country economic status (Table 4).

Weight-for-age (z-score) was reported as an outcome in seven articles, four of which were included in the meta-analysis [28,30,35,36]. The change in weight-for-age z-scores across the intervention were not different between the fortified milk and control groups (MD 0.08 z-score [95% CI −0.01, 0.18], random-effects model, *p* = 0.09; 4 studies, *n* = 1262, Figure 2). Again, there was high heterogeneity between individual studies (I^2^ = 73%, *p* = 0.01, Figure 2). Considerable heterogeneity remained after removal of the only study that favoured the control condition, and the overall difference between fortified milk and control groups was similar (MD 0.12 z-score [95% CI 0.05, 0.20], random effects model, I^2^ = 53%, *p* = 001; *n* = 1419). 

Length-for-age and weight-for-length z-scores were reported as an outcome in six studies, three of which were included in the meta-analysis [30,35,36]. There was no significant difference in the change in length z-scores between the fortified milk and control groups (MD −0.02 z-score [95% CI −0.25, 0.21], random effects model, *p* = 0.88; 3 studies, *n* = 1169). In contrast, the change in weight-for-length z-score was higher in the fortified milk groups compared to controls in the pooled analysis (MD 0.10 z-score [95% CI 0.02, 0.17], random effects model, *p* = 0.01; three studies, *n* = 1169). There was considerable heterogeneity between studies in the case of length-for-age (I^2^ = 94%; *p* < 0.00001, Figure 2), whereas the weight-for-length outcome exhibited no heterogeneity between studies (*p* = 0.61, I^2^ = 0%; Figure 2). One study [41] reported changes in weight- and length-for-age and weight-for-length z-scores at the end of the intervention period between groups separately by sex and found no differences between the fortified milk and control groups in either males or females. 

Head circumference (cm) was reported in two articles [28,29], neither of which reported a significant effect of the fortified milk intervention on this parameter. 

#### 3.2.2. Body Composition Outcomes

BMI (kg/m^2^) was reported as an outcome in four articles, three of which were included in the meta-analysis [28,30,32]. There was no difference in BMI changes pre- and post-intervention between the fortified milk and control groups (MD 0.29 kg/m^2^ [95% CI −0.07, 0.66], random effects model, *p* = 0.11; 3 studies, *n* = 1337). There was considerable heterogeneity between studies (*p* = 0.11, I^2^ = 55%; Figure 2). 

Waist circumference and waist-to-height ratio were reported in one study [30], which found no difference in these parameters between the fortified milk and control groups either at the end of the intervention period or in the changes in these parameters during the intervention period. 

Mid-upper arm circumference and skinfold thickness (measured at two sites: subscapular and triceps) were reported in one study [29], which compared an iron-fortified “young child formula” with a lower iron formulation and cow’s milk. This study found no effect of the iron-fortified formula on either parameter when compared to either the lower iron formula or cow’s milk groups. 

One study [45] reported changes in percent body fat, fat mass, fat-free mass, and fat mass index, measured by bioelectrical impedance at the end of a 12-month intervention period. The study found that body fat (%), fat mass (kg), and fat mass index (kg/m^2^) at the end of the intervention period were lower in the group that received the fortified young child formula (Growing Up Milk Lite) when compared to the control (cow’s milk) group, whereas there were no differences in these parameters between groups at baseline. There were no significant differences in fat-free mass between the intervention and control groups or changes in this measure over the duration of the intervention. 

### 3.3. Effect of Formula on Biochemical Outcomes

#### 3.3.1. Iron

A number of biochemical markers were utilised to assess iron status in the infants before, during, and following the intervention, including Hb, ferritin, transferrin, and soluble transferrin receptor concentrations. 

**Figure 3:**
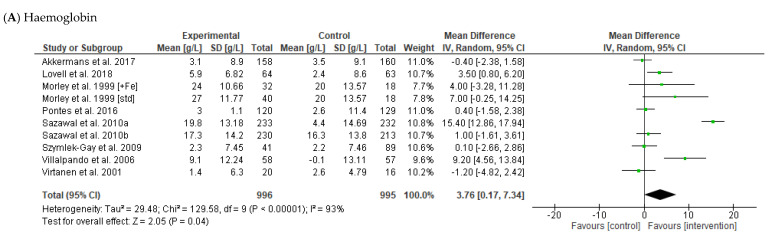

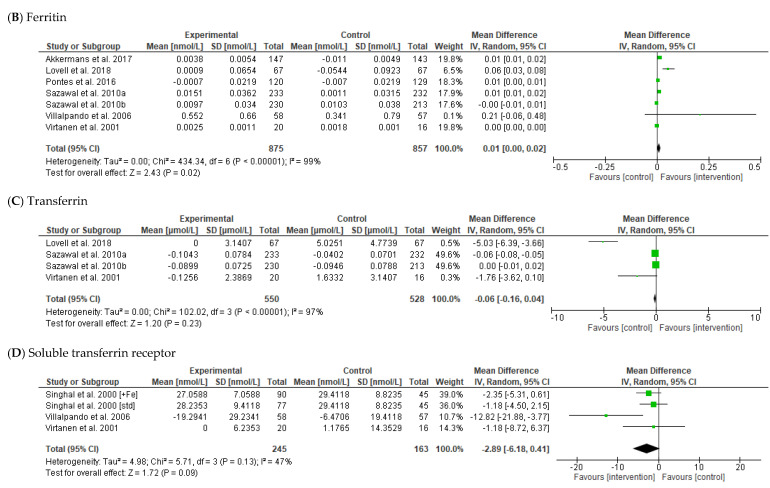
Forest plot displaying the effect of fortified milk (intervention) compared with control milk on mean difference in (**A**) haemoglobin (g/L) [29,31,33,34,35,36,37,41,43]; (**B**) ferritin (nmol/L) [31,33,34,35,36,37,41]; (**C**) transferrin (μmol/L) [31,34,36,37]; (**D**) soluble transferrin receptor (nmol/L) [34,37,46]. Note: The study-specific mean differences and 95% confidence intervals are represented by the green squares and horizontal lines, respectively. The size of the square represents the weight given to each study in the meta-analysis. The shaded diamond represents the pooled mean difference, and the width of the diamond represents the pooled 95% confidence interval.

Haemoglobin (g/L) was reported in eleven articles, nine of which were included in the meta-analysis [29,31,33,34,35,36,37,41,43]. This analysis indicated that infants receiving fortified milks had a significantly greater mean change in Hb concentration across the intervention period compared to the control group (MD 3.76 g/L [95% CI 0.17, 7.34], random effects model, *p* = 0.04; 9 studies, *n* = 1991). There was considerable heterogeneity between studies for this outcome measure (I^2^ = 93%, *p* = 0.04; Figure 3). The changes in Hb observed were not significant in subgroup analyses of intervention length and country economic status (Table 4).

Serum ferritin (nmol/L) was reported in 11 articles, seven of which were included in the meta-analysis [31,33,34,35,36,37,41]. When the data were pooled, participants in the intervention groups had a statistically significant greater mean change in serum ferritin concentrations at the end of the intervention in infants assigned to receive fortified milk compared to the control/comparator groups (MD 0.01 nmol/L [95% CI 0.00, 0.02], random effects model, *p* = 0.02; 7 studies, *n* = 1732). There was substantial heterogeneity between the included studies (I^2^ = 99%, *p* < 0.00001; Figure 3). Subgroup analyses indicated that the greater increase in serum ferritin in the fortified milk vs control group was significant for studies >6 months in duration (MD 0.01 nmol/L [95% CI 0.00, 0.02], *p* = 0.03; four studies, *n* = 1291) but not in shorter studies (Table 4). In addition, the difference in serum ferritin between fortified milk and controls was significant for studies conducted in higher-income countries (MD 0.01 nmol/L [95% CI 0.00, 0.03], *p* = 0.02; 3 studies, *n* = 460), but not those conducted in lower-income countries (Table 4).

Serum transferrin (μmol/L) was reported in four articles, all of which were included in the meta-analysis [31,34,35,36]. There was no difference in the change in serum transferrin concentration between intervention and control groups (MD −0.06 μmol/L [95% CI −0.16, 0.04], random effects model, *p* = 0.23; 4 studies, *n* = 1078). Again, there was a high degree of heterogeneity between studies (I^2^ = 97%, *p* < 0.00001; Figure 3). 

Soluble transferrin receptor (μmol/L) was reported in six articles, three of which were included in the meta-analysis [34,37,46]. There was no significant difference in soluble transferrin receptor concentration at the end of the intervention period between infants receiving fortified milks compared to control (MD −2.89 μmol/L [95% CI −6.18, 0.41], random effects model, *p* = 0.09; 4 studies, *n* = 408; Figure 3). 

#### 3.3.2. Other Biochemical Markers

Vitamin D (serum 25-hydroxyvitamin D) was measured in three studies [31,33,48]. Two studies [31,33] reported significant increases in serum vitamin D concentrations during the intervention period in infants assigned to the fortified milk groups, which were greater than those in the control group. In contrast, Houghton et al. [48] found no difference in either vitamin D concentrations at the end of the intervention period, or changes in vitamin D concentrations from baseline, between infants assigned to the fortified milk group compared to controls. 

Zinc (plasma) was measured in four studies [35,36,41,47], none of which reported any effect of the fortified milk on zinc concentrations either at the end of the intervention period or the change in zinc concentrations from baseline. 

One study [49] measured urinary iodine concentrations at baseline and at the end of the intervention period, and found that infants who were provided with a fortified milk drink containing 21.1 µg iodine/100 g (intervention group) had higher urinary iodine concentrations at the end of the intervention period compared to those who were provided with non-fortified milk (containing 5.6 µg/100 g of iodine; MD 85 µg/L [95% CI 58, 113], *p* < 0.001).

### 3.4. Dietary Intake

Energy intake was reported across six articles that comprised four studies (Supplementary Table S3). One study [46] reported higher energy intake at the end of the study in both intervention groups (fortified milks with different contents of iron) compared with that of the control group (*p* = 0.025). There were no differences between intervention and control groups in energy intake reported in the other three studies [35,44,49]. Two studies [35,44] reported changes in macronutrient intakes during the study period but found no difference in intakes of either fat or carbohydrates between the fortified milk and control groups at the end of the study period. 

Findings for protein intakes were mixed. Sazawal et al. [35] reported higher intakes of protein in the fortified milk group compared with the control group during the intervention period, whereas Lovell et al. [44] reported the opposite effect. This was likely attributable to the different compositions of milk prescribed (intervention: ‘lite’ growing-up milk, 1.7% protein; and control: cow’s milk, 3.1% protein) in the Lovell et al. study. In the same trial, intakes of vitamins C and D were higher and intakes of vitamin B12 were lower in the fortified milk group compared to controls.

Iron intake was reported in four studies [34,35,43,44], all of which reported higher intakes of iron in the intervention groups compared with the control at the end-study. Virtanen et al. [34] further investigated the intake of iron from different foods and found almost 50% of dietary iron intake came from the milk beverage in the fortified milk group, whereas red meat was the primary source of iron in the control group, and only approximately 8% was obtained from the control milk beverage.

### 3.5. Sensitivity Analyses

When sensitivity analysis was performed, results were not different from the pooled data when studies with a ‘high risk’ of bias [34,37] were excluded from any outcome. Sensitivity analysis was also conducted for studies with imputed data. Results were not different from the pooled data for weight or length/height outcomes. However, the differences in Hb and ferritin between groups were no longer significant when these studies were excluded (Table 4).

### 3.6. Publication Bias

Funnel plots were examined to graphically inspect for the presence of publication bias in the results of the meta-analysis. Generally, studies were spread on either side of the mean effect and standard errors of intervention effect were similar across studies (Supplementary Figure S2), suggesting the scatter is due to sampling variation, rather than publication bias. However, the use and appropriate interpretation of funnel plots have well-documented limitations [50], and Sterne and colleagues [51] recommend tests for funnel plot asymmetry should not be used when there are <10 studies included in the meta-analysis, and studies are of similar size. Therefore, the authors recommend caution when interpreting the results; and publication bias may be present.

## 4. Discussion

Adequate nutrition and appropriate feeding are critical for healthy growth and development. However, when transitioning to weaning foods, young children may be at risk of nutrient deficiencies. The purpose of this study was to review the effects of fortified milk consumption compared with control milk on health outcomes in young children. There were some differences in measures of growth (weight and weight-for-length) and biochemistry (haemoglobin and serum ferritin) favouring the consumption of fortified milk, and these changes appear to be independent of dietary intake from food and other sources. However, there was no effect of the intervention on most other outcomes included in the review.

Regarding parameters of growth, findings from the meta-analysis showed that the consumption of fortified milk by young children was associated with a greater weight gain than those consuming an equivalent amount of non-fortified/cow’s milk (mean difference = 0.14 kg). There were no significant findings for other anthropometric measures. These findings are similar to those reported by Matsuyama et al. [17] who reported a small but significant effect of fortified milk on growth outcomes (mean difference = 0.17 kg body weight) in their meta-analysis. When subgroup analyses were conducted to investigate the effect of intervention length on changes in body weight, only studies longer than six months showed a significant mean difference in body weight. Considering weight gain from 12 to 24 months of age is expected to be approximately 2–3 kg, this suggests that the effect of consuming young child formula is unlikely to have a clinically significant impact on growth in the majority of healthy infants, but is also unlikely to have an adverse effect on growth [52,53]. These findings also indicate that young child formula consumption does not lead to any clinically relevant increase in the rate of weight gain, which is important since accelerated growth during this period has been associated with an increased risk of obesity later in life [54]. The effect of fortified milk consumption on weight gain was restricted to low-income countries in subgroup analysis; suggesting that the effect of young child formula may be related to a child’s nutritional status at baseline and may play a role in supporting weight gain in children at higher risk of inadequate caloric intakes, which is more prevalent in low-income regions.

Micronutrient deficiencies are estimated to affect more than two billion people globally [55]. Young child formula and fortified milks contained added micronutrients, including iron and vitamin D, and studies have included biochemical measures to assess the effect of fortified milks on nutritional status. Iron was most frequently investigated, likely due to the known inadequacy of intake of iron from foods in this population [6,31,56,57,58,59]. The meta-analysis showed there was a significant mean difference in change in the baseline to endpoint values for Hb (3.76 g/L) and ferritin (0.01 nmol/L), suggesting that fortified milk was associated with improved iron status compared to the control milks. These findings suggest that, when consumed as part of a diet, iron-fortified milk may be more effective at improving the iron status of young children compared to cow’s milk. However, the difference in ferritin was small, and unlikely to be clinically relevant for most infants, but it is unknown if it could be helpful in some circumstances, such as to supplement iron intake in the diet, which is often challenging in this age group. Between the ages of 6–24 months, infants and toddlers experience rapid growth, and increased iron requirements to aid in development [60]. During this time, iron requirements are higher than at any other life stage [60]. Iron deficiency can result in iron deficiency anaemia, which can have negative and potentially irreversible impacts on short- and long-term neuro-behavioural development [6]. Reviews of studies on iron supplementation have shown mixed results, ranging from beneficial, to null, to detrimental effects on growth (length). Reaching adequate iron intake through iron-fortified whole foods is encouraged, however, young child formulas may be one strategy to increase iron intake in situations where obtaining it from the diet alone is problematic [19]. 

The studies included in this review used different indicators of iron status and anaemia (haemoglobin, serum ferritin, and serum transferrin) [61,62] as well as different concentrations of iron fortification in the intervention milks. An unexpected finding was that, in subgroup analysis, greater serum ferritin concentrations in the fortified milk groups was observed for higher-income, but not lower-income, countries. One possible explanation is that the fortified milks provided to infants had a higher iron content than those used in lower-income countries and/or was more bioavailable, either due to the form of iron used, presence of factors to increase (e.g., vitamin C) or inhibit (e.g., phytates) iron absorption and/or greater diversity of the background diet. However, given the relatively small number of studies, and differences between studies on the composition of fortified milks provided and the duration of the interventions, it is difficult to draw clear conclusions.

Young children’s feeding practices are influenced by a range of factors including socio-cultural and economic factors, as well as access and availability of foods and food products, and advice from healthcare professionals [63]. There have been concerns from parents, advisory groups, paediatricians, and other health professionals that the inclusion of fortified milk in young children’s diets may affect diet quality. It has been suggested, for example, that parents who provide fortified milk to their children may feel a sense of ‘nutritional security’, which may in turn impact dietary variety, displace energy intake, or inadvertently limit foods offered to young children [12,43]. Most studies included in this review did not report any changes in total energy or intakes of fat and carbohydrate from baseline, suggesting that the addition of milk (fortified or control) did not alter children’s usual intakes. However, it is not known if children’s intakes from food sources were displaced by the addition of milk. There were some differences reported in the intakes of protein and specific micronutrients in infants consuming fortified milks, which is not unexpected given the different compositions of the fortified milk products compared to standard cow’s milk. It is also important to note that dietary intake was assessed in relatively few studies, and those that did report on diet generally focused on overall energy, or single-nutrient intake (such as iron or zinc), with few describing multiple-nutrient intakes. Therefore, based on the limited available evidence, the effect of fortified milk on dietary intake is unclear. Estimates from the Australian Feeding Infants and Toddlers Study (OzFITS) [12], reported that one-fifth of young children consumed formula or toddler milks (on the day of the food record), contributing to 18–23% of total energy, thus, understanding the relationship between fortified milk consumption and dietary intake from other sources will be an important area for future study.

This review provides a robust evaluation of the current evidence evaluating the effect of fortified milk consumption on health outcomes in young children. Strengths of this review include its prospective registration, inclusion only of randomised controlled trials, and comprehensive search strategy. However, despite its strengths, some limitations must be acknowledged. Articles not published in the English language were excluded, which may have introduced bias [64]. A common constraint in meta-analyses is heterogeneity of the included studies [65]. In the current meta-analysis, there was substantial heterogeneity between studies, therefore, findings reported herein should be interpreted with caution. It is also important to acknowledge the potential for dependence between effects included in the meta-analysis, which can occur when a study’s research design includes two intervention groups compared with the same control group, or when a study included in the meta-analysis uses more than one outcome measure, to have impacted the findings. While efforts were made to reduce effects associated with dependence in studies using the same control group by splitting the shared group into two groups with smaller (halved) sample sizes, the small number of studies made it difficult to adequately control for dependence effects for studies with multiple outcome measures. Finally, due to the limited available evidence, publication bias could not properly be assessed, and thus cannot be excluded as a factor contributing to the results. 

Nutrition practices established during infancy and early childhood set the foundation for eating patterns in later years. While obtaining the appropriate balance of nutrients from dietary sources is the optimal approach, the challenges of meeting dietary guidelines are recognised across all age groups, suggesting that supplemental sources of specific macro- or micronutrients may be warranted in some circumstances, particularly where access to safe and nutritious food is limited or other barriers to increasing dietary diversity persist despite persistent attempts at a “food first approach”. This systematic review and meta-analysis found that consuming fortified milk may improve some aspects of growth and nutritional status in young children, particularly in study populations where children were at risk of undernutrition. It is important to note, however, that there is a wide range of these products available, with variable compositions, so the selection of an appropriate product is important and should be guided by an appropriately qualified health professional. In addition, the long-term effects of sustained use of young child formula were beyond the scope of this review and warrant further research. With an increasing number of parents utilising young child formula, it will be important to better understand their motivation for doing so, so that appropriate education and support can be provided.

## Figures and Tables

**Table 1:** Inclusion and exclusion criteria.

Population	Apparently Healthy Children Aged 9 to 48 * Months at Trial Commencement
Intervention/Exposure	Fortified milk or formula with added macronutrients, micronutrients and/or prebiotics, probiotics, or synbiotics
Comparison	Non-fortified milk or formula
Outcomes	Growth parameters including body size (e.g., weight, length (/height), body mass index, head circumference) Body composition (including skinfold thickness, waist circumference, body fat) Biochemical markers (including vitamin D, zinc, iron-related measures (serum ferritin, serum transferrin, haemoglobin) Dietary intake (energy, macro-, and micro-nutrients)
Study design	Randomised controlled trials
Exclusion criteria	Studies published prior to 1990 Studies published in non-English language Animal studies In vitro studies Studies in adolescents and adults Acute studies (<3-month intervention period) Observational studies, review articles, conference abstracts, protocol papers

Note: *, initially, the eligible age was set at 12–36 months, however, due to the wide age ranges included in studies, this age limitation was not feasible. Studies that enrolled children from 9 months and up to 48 months of age were included in the review if >50% of the intervention was conducted during 12–36 months of age.

**Table 2:** Characteristics of included studies.

Author, Year [Reference]	Country	Study Design	Intervention Length	Sample Size (Enrolled)	Sex (*n*, % Male)	Age Range Recruited	Co-Conditions	Quality Assessment
**Akkermans et al., 2017** [33]	The Netherlands, United Kingdom, Germany	Randomised, double-blinded controlled trial	20 weeks	*n* = 318	*n* = 180, 57%	12–36 months	18% of sample had anaemia	Some concerns
**Chatchatee et al., 2014** [32]	Malaysia, the Netherlands, Poland, Portugal, Thailand	Randomised, double-blinded controlled trial	12 months	*n* = 804	Mixed sex, distribution NR	11–29 months	NR	Some concerns
**Firmansyah et al., 2011** [28]	Indonesia	Randomised, double-blinded controlled trial	12 months ^§^	*n* = 393	*n* = 203, 52%	12 months	NR	Some concerns
**Pontes et al., 2016** [41]	Brazil	Randomised, double-blinded controlled trial	28 weeks	*n* = 256	Mixed sex, distribution NR	12–48 months	NR	Some concerns
**Rivera et al., 2010** [42]	Brazil	Double-blinded, group randomised effectiveness trial	12 months	*n* = 795	Mixed sex, distribution NR	12–30 months	~43% of sample had anaemia	Some concerns
**Sazawal et al., 2010a** [35]	India	Randomised, double-blinded controlled trial	12 months	*n* = 633	Mixed sex, distribution NR	12–36 months	~54% of sample had iron deficiency anaemia ~47% were stunted ~5% were wasted ~16% were wasted and stunted	Some concerns
**Sazawal et al., 2010b** [36]	India	Randomised, double-blinded controlled trial	12 months	*n* = 624	Mixed sex, distribution NR	12–36 months	~56% of sample had iron deficiency anaemia ~47% were stunted ~5% were wasted ~16% were wasted and stunted	Low risk of bias
**Villalpando et al., 2006** [37]	Mexico	Randomised, double blinded clinical trial	6 months	*n* = 130	*n* = 65, 50%	10–30 months	~36% of sample had anaemia	High risk of bias
**Virtanen et al., 2001** [34]	Sweden	Randomised, double blinded controlled trial	6 months	*n* = 54	*n* = 21, 39%	12 months	NR	High risk of bias
**Studies with more than one article**								
**GUMLi trial**								
**Wall et al., 2019** [30] Lovell et al., 2018 [31] ^ⱡ^ Lovell et al., 2019 [44] ^ⱡ^ Lovell et al., 2021 [45] ^ⱡ^	Australia, New Zealand	Randomised, double-blinded controlled trial	12 months	*n* = 160	*n* = 85, 53%	12 months ± 2 weeks	NR	Some concerns Some concerns Some concerns Some concerns
**Large UK trial**								
**Morley et al., 1999** [29] Singhal et al., 2000 [46] ^ⱡ^	United Kingdom	Randomised, blinded controlled trial	9 months	*n* = 493	*n* = 257, 52%	9 months	NR	Low risk of bias Low risk of bias
**Toddler Food Study**								
**Szymlek-Gay et al., 2009** [43] * Morgan et al., 2010 [47] ^ⱡ^,* Houghton et al., 2011 [48] ^ⱡ^,* Szymlek-Gay et al., 2020 [49] ^ⱡ^,*	New Zealand	Randomised, blinded placebo-controlled trial	5 months	*n* = 135	*n* = 62, 46%	12–20 months	NR	Low risk of bias Some concerns Some concerns Some concerns

Note: **Bold text** indicates primary research study; ^ⱡ^, indicates the secondary analysis of primary study; *, indicates study had an additional intervention or control arm not extracted; ^§^, intervention duration was 12 months, but change in relevant outcome was reported at 4 months. Abbreviations: *n*, number; NR, not reported.

**Table 3:** Intervention data and outcome variables.

First Author, Year [Reference]	Study Outcomes Included in the Review	Intervention Condition/s				Control Condition				Adherence Check (Method)
		Milk Type	Amount of Milk Prescribed	Amount of Milk Consumed	Completers (*n*, %)	Milk Type	Amount of Milk Prescribed	Amount of Milk Consumed	COMPLETERS (*n*, %)	
**Akkermans et al., 2017** [33]	Biochemical: Fe (Hb, SF), Fe deficiency, Fe deficiency anaemia, Vit D	Young child formula	>150 mL/d	NR	114 (72%)	Cow’s milk	>150 mL/d	NR	113 (71%)	Parent phone calls
**Chatchatee et al., 2014** [32]	Growth: weight, length, BMI	Int 1 = Growing Up Milk (GUM) with added prebiotics and LCPUFAs Int 2 = GUM	400 to 750 mL/d	Int 1 = 529 mL/d Int 2 = 527 mL/d	Int 1 = 348 (90%) Int 2 = 349 (92%)	Cow’s milk	400 to 750 mL/d	527 mL/day	33 (100%)	Study diary
**Firmansyah et al., 2011** [28] **^§^**	Growth: weight, length, BMI, WFA	Synbiotic Milk (BL999, LPR and prebiotics) with LCPUFA	400 mL/d (200 mL twice, daily)	426 ± 25.9 mL/d	161 (81%)	Cow’s milk	400 mL/d (200 mL twice, daily)	426 ± 31.2 mL/d	153 (79%)	NR
**Pontes et al., 2016** [41]	Growth: WFA, length, LFA Biochemical: Fe (Hb, SF), Zn	Cow’s milk-based beverage fortified with DHA, prebiotics (PDX, GOS, β-glucan) and other nutrients	720 mL/d (240 mL thrice daily)	12–24 months of age: 504 mL/d 25–48 months of age: 498 mL/d	120 (96%)	Cow’s milk-based beverage	720 mL/d (240 mL thrice daily)	12–24 months of age: 531 mL/d 25–48 months of age: 547 mL/d	129 (98%)	NR
**Rivera et al., 2010** [42]	Growth: WFA, LFA, WFL (data not shown) Biochemical: Fe (Hb, SF, sTfR), anaemia	Fortified milk formula	400 mL/d (200 mL twice daily)	611 mL/d ^ⱡ^	371	Non-fortified milk formula	400 mL/d (200 mL twice daily)	609 mL/d ^ⱡ^	213	Field workers conducted weekly surveys
**Sazawal et al., 2010a** [35]	Growth: weight, WFA, length, LFA, WFL Biochemical: Fe (Hb, SF, Tf) Fe deficiency, Fe deficiency anaemia, Zn Nutrient intake: energy, protein, fat, CHO, Fe, Zn	Milk powder fortified with Zn, Fe, Se, Cu, Vits A, C and E	96 g (32 g single-serving sachet thrice daily)	NR	289 (91%)	Cow’s milk powder	96 g (32 g single-serving sachet thrice daily)	NR	281 (89%)	Household milk assistants collected weekly data on compliance
**Sazawal et al., 2010b** [36]	Growth: weight, WFA, length, length, LFA, WFL Biochemical: Fe (Hb, SF, Tf), Fe deficiency, Fe deficiency anaemia, Zn	Milk powder fortified with probiotic B lactis HN019 and prebiotic oligosaccharides	96 g (32 g single-serving sachet thrice daily)	NR	296 (95%)	Cow’s milk powder	96 g (32 g single-serving sachet thrice daily)	NR	285 (91%)	NR
**Villalpando et al., 2006** [37]	Biochemical: Fe (Hb, SF, sTfR)	Milk powder fortified with Fe and other micronutrients	400 mL/d (200 mL twice daily)	482 mL/d	58 (88%)	Cow’s milk powder	400 mL/d (200 mL twice daily)	529 mL/d	57 (88%)	A field worker observed and registered milk intake
**Virtanen et al., 2001** [34]	Growth: weight Biochemical: Fe (Hb, SF, Tf, sTfR), iron deficiency, Fe deficiency anaemia Nutrient intake: Fe	Iron-fortified (lower protein) cow’s milk	Ad lib	445 mL/d	20	Cow’s milk	Ad lib	562 mL/d	16	NR
**Studies with more than one article**										
**GUMLi trial**										
**Wall et al., 2019** [30]	Growth: weight, WFA, length, LFA, WFL, BMI, BMIFA, WC, waist-height ratio	GUM Lite (GUMLi) with reduced protein, synbiotics, and micronutrients added	300 mL/d	NR	67 (84%)	Cow’s milk	300 mL/d	NR	67 (84%)	Monthly telephone or face-to-face questionnaire with parents or guardians
Lovell et al., 2018 [31]	Biochemical: Fe (Hb, SF, Tf, sTfR), iron deficiency, Fe deficiency anaemia, Vit D Nutrient intake: Fe									
Lovell et al., 2019 [44]	Nutrient intake: energy, protein, fat, CHO, Fe									
Lovell et al., 2021 [45]	Growth: weight, WFA, length, LFA, WFL, BMI, BMIFA, body fat %, FFM, FM, FMI Nutrient intake: energy, protein									
**Large UK trial**										
**Morley et al., 1999** [29]	Growth: weight, length, skinfold, HC, MUAC Biochemical: Fe (Hb, SF, sTfR)	Int 1 = Fe-fortified formula (12 mg/L as ferrous sulfate) Int 2 = Standard formula (0.9 mg/L of Fe)	Ad lib	NR	*n*_(int 1)_ = 135 (82%) *n*_(int 2)_ = 133 (82%)	Cow’s milk	Ad lib	NR	*n*_(con)_ = 160 (96%)	Nurse visit at mid- and end-intervention (12 and 15 months, respectively)
Singhal et al., 2000 [46]	Growth: weight. Biochemical: Fe (Hb, SF, sTfR) Nutrient intake: energy									
**Toddler Food Study**										
**Szymlek-Gay et al., 2009** [43] *	Biochemical: Fe (Hb, SF, sTfR) Nutrient intake: Fe	Iron, zinc, vitamin C and vitamin D, iodine fortified milk (lower protein)	~370 mL/d	407	41 (91%)	Non-fortified milk (with required vit D and A)	~370 mL/d	445	81 (90%)	Parents kept adherence records
Morgan et al., 2010 [47] *	Biochemical: Zn Nutrient intake: energy, fibre, Zn, Ca									
Houghton et al., 2011 [48] *	Biochemical: Vit D									
Szymlek-Gay et al., 2020 [49] *	Biochemical: Iodine Nutrient intake: energy, iodine									

Note: **Bold text** indicates primary research study; *, indicates study had an additional intervention/control arm not extracted; ^§^, intervention duration was 12 months, but a change in relevant outcome was reported at 4 months; ^ⱡ^, reported intake includes all milk consumed (i.e., from usual diet plus prescribed). Abbreviations: *n*, number; Fe, Iron; Hb, haemoglobin; SF, Serum Ferritin; Vit, vitamin; ml/day, millilitre per day; NR, not reported; BMI, body mass index; LCPUFAs, long-chain polyunsaturated fatty acids; WFA, weight-for-age; BL999, Bifidobacterium longum; LPR, Lactobacillus rhamonosus; LFA, length-for-age; Zn, zinc; DHA, Docosahexaenoic acid; PDX, polydextrose; GOS, Galacto-oligosaccharides; WFL, weight-for-length; sTfR, Serum Transferrin Receptor; Tf, transferrin; CHO, carbohydrate; Se, selenium; Cu, copper; ad lib, ad libitum; BMIFA, body mass index-for-age; WC, waist circumference; FFM, fat-free mass; FM, fat mass; FMI, fat mass index; HC, head circumference; MUAC, Mid-upper arm circumference; Ca, calcium.

**Table 4:** Meta-analysis results of the overall effect (pooled data), subgroup, and sensitivity analyses of selected outcomes.

	Weight (kg)		Length/Height (cm)		Hb (g/L)		Ferritin (nmol/L)		Transferrin (μmol/L)	
	*n* Participants (*n* Studies) [Reference]	MD [95% CI] (Overall Effect *p*-Value; I^2^ %)	*n* Participants (*n* Studies) [Reference]	MD [95% CI] (Overall Effect *p*-Value; I^2^ %)	*n* Participants (*n* Studies) [Reference]	MD [95% CI] (Overall Effect *p*-Value; I^2^ %)	*n* Participants (*n* Studies) [Reference]	MD [95% CI] (Overall Effect *p*-Value; I^2^ %)	*n* Participants (*n* Studies) [Reference]	MD [95% CI] (Overall Effect *p*-Value; I^2^ %)
Pooled result	*n* = 2829 (7 studies) [28,29,30,32,34,35,36]	0.14 [0.06, 0.21] (*p* = 0.0003; I^2^ = 20%)	*n* = 2791 (6 studies) [28,29,30,32,35,36]	−0.05 [−0.41, 0.32] (*p* = 0.81; I^2^ = 80%)	*n* = 1991 (9 studies) [29,31,33,34,35,36,37,41,43]	3.76 [0.17, 7.34] (*p* = 0.04; I^2^ = 93%)	*n* = 1732 (7 studies) [31,33,34,35,36,37,41]	0.01 [0.00, 0.02] (*p* = 0.02; I^2^ = 99%)	*n* = 1078 (4 studies) [31,34,35,36]	−0.06 [−0.16, 0.04] (*p* = 0.23; I^2^ = 97%)
Subgroup analyses										
Country economic status *										
Lower-income countries	*n* = 1419 (3 studies) [28,35,36]	0.16 [0.08, 0.23] (*p* < 0.0001; I^2^ = 16%)	*n* = 1419 (3 studies) [28,35,36]	0.23 [−0.25, 0.70] (*p* = 0.36; I^2^ = 89%)	*n* = 1272 (4 studies) [35,36,37,41]	6.44 [−1.24, 14.12] (*p* = 0.10; I^2^ = 97%)	*n* = 1272 (4 studies) [35,36,37,41]	0.01 [−0.00, 0.02] (*p* = 0.11; I^2^ = 75%)	N/A ^§^	
Higher-income countries	*n* = 609 (3 studies) [29,30,34]	0.06 [−0.15, 0.27] (*p* = 0.57; I^2^ = 51%)	N/A ^§^		*n* = 719 (5 studies) [29,31,33,34,43]	1.18 [−0.83, 3.19] (*p* = 0.25; I^2^ = 51%)	*n* = 460 (3 studies) [31,33,34]	0.01 [0.00, 0.03] (*p* = 0.02; I^2^ = 100%)	N/A ^§^	
Intervention length										
≤6 months	N/A ^§^		N/A ^§^		*n* = 599 (4 studies) [33,34,37,43]	1.50 [−1.95. 4.95] (*p* = 0.39; I^2^ = 80%)	*n* = 441 (3 studies) [33,34,37]	0.01 [−0.01, 0.02] (*p* = 0.24; I^2^ = 100%)	N/A ^§^	
>6 months	*n* = 2400 (5 studies) [29,30,32,35,36]	0.15 [0.06, 0.25] (*p* = 0.001; I^2^ = 31%)	*n* = 2398 (5 studies) [29,30,32,35,36]	0.05 [−0.30, 0.41] (*p* = 0.77; I^2^ = 73%)	*n* = 1391 (5 studies) [29,31,35,36,41]	5.19 [−0.32, 10.69] (*p* = 0.07; I^2^ = 95%)	*n* = 1291 (4 studies) [31,35,36,41]	0.01 [0.00, 0.02] (*p* = 0.03; I^2^ = 86%)	*n* = 1042 (3 studies) [31,35,36]	−0.05 [−0.15, 0.04] (*p* = 0.27; I^2^ = 98%)
Sensitivity analyses										
Data treatment										
Excluding studies with imputed data	*n* = 1863 (4 studies) [32,34,35,36]	0.17 [0.10, 0.24] (*p* < 0.00001; I^2^ = 0%)	*n* = 1827 (3 studies) [32,35,36]	0.39 [0.05, 0.72] (*p* = 0.02; I^2^ = 53%)	*n* = 733 (4 studies) [33,34,41,43]	−0.11 [−1.29, 1.07] (*p* = 0.86; I^2^ = 0%)	*n* = 690 (4 studies) [32,33,37,41]	0.01 [−0.00, 0.02], *p* = 0.18; I^2^ = 99%)	N/A ^§^	
Risk of bias										
Excluding studies with high risk of bias [34,37]	*n* = 2793 (6 studies) [28,29,30,32,35,36]	0.14 [0.06, 0.22] (*p* = 0.0007; I^2^ = 28%)	N/A ^ⱡ^		*n* = 1840 (7 studies) [29,31,33,35,36,41,43]	3.74 [−0.35, 7.84] (*p* = 0.07; I^2^ = 94%)	*n* = 1581 (5 studies) [31,33,35,36,41]	0.01 [0.00, 0.02] (*p* = 0.002; I^2^ = 89%]	*n* = 1042 (3 studies) [31,35,36]	−0.05 [−0.15, 0.04] (*p* = 0.27; I^2^ = 98%)

Note: only outcomes with three or more studies in both subgroups are presented; *, one study [32] was conducted across several countries (Malaysia, the Netherlands, Poland, Portugal, and Thailand) and was excluded from the subgroup analysis of country economic status; ^§^, subgroup/sensitivity analyses not conducted as there were fewer than three studies applicable; ^ⱡ^, sensitivity analysis not conducted as there were no studies included in the meta-analysis with a high risk of bias. Abbreviations: MD, mean difference; CI, confidence interval; N/A, not applicable.

## Data Availability

Data is contained within the article or supplementary material.

## Supplementary Materials

The following supporting information can be downloaded at: https://www.mdpi.com/article/10.3390/nu14235060/s1, Table S1: PRISMA Checklist 2020; Table S2: Literature Search Strategy; Table S3: Summary of outcome measures reported across the articles and included in the review; Figure S1: Quality assessment of the included studies using the Cochrane Risk of Bias 2.0 Tool; Figure S2: Funnel plots of intervention effect estimates (mean difference) from individual studies against the standard error of the effect estimate.
