# Supplementary material for: Effect of Fortified Formula on Growth and Nutritional Status in Young Children: A Systematic Review and Meta-Analysis
Source: Nutrients. 2022 Nov 28;14(23):5060. doi: 10.3390/nu14235060 (PMC9737957; doi:10.3390/nu14235060)
Supplement: Supplementary file 1 [file nutrients-14-05060-s001.zip › nutrients-2023167-supplementary.pdf]

Supplementary Material associated with the submission *'Effect of fortified formula on growth and nutritional status in young children: a systematic review and meta-analysis'*

Table S1 PRISMA Checklist 2020

Section and Topic	Item #	Checklist item	Location where item is reported
**TITLE**			
Title	1	Identify the report as a systematic review.	Title (p.1)
**ABSTRACT**			
Abstract	2	See the PRISMA 2020 for Abstracts checklist.	p.1
**INTRODUCTION**			
Rationale	3	Describe the rationale for the review in the context of existing knowledge.	pp.1-2
Objectives	4	Provide an explicit statement of the objective(s) or question(s) the review addresses.	p.2
**METHODS**			
Eligibility criteria	5	Specify the inclusion and exclusion criteria for the review and how studies were grouped for the syntheses.	pp.3-4
Information sources	6	Specify all databases, registers, websites, organisations, reference lists and other sources searched or consulted to identify studies. Specify the date when each source was last searched or consulted.	p.3
Search strategy	7	Present the full search strategies for all databases, registers and websites, including any filters and limits used.	Supp. Mat. Table S2
Selection process	8	Specify the methods used to decide whether a study met the inclusion criteria of the review, including how many reviewers screened each record and each report retrieved, whether they worked independently, and if applicable, details of automation tools used in the process.	pp. 3-4
Data collection process	9	Specify the methods used to collect data from reports, including how many reviewers collected data from each report, whether they worked independently, any processes for obtaining or confirming data from study investigators, and if applicable, details of automation tools used in the process.	pp.3-5
Data items	10a	List and define all outcomes for which data were sought. Specify whether all results that were compatible with each outcome domain in each study were sought (e.g. for all measures, time points, analyses), and if not, the methods used to decide which results to collect.	pp.3-5
	10b	List and define all other variables for which data were sought (e.g. participant and intervention characteristics, funding sources). Describe any assumptions made about any missing or unclear information.	pp.3-5
Study risk of bias assessment	11	Specify the methods used to assess risk of bias in the included studies, including details of the tool(s) used, how many reviewers assessed each study and whether they worked independently, and if applicable, details of automation tools used in the process.	pp.3-5
Effect measures	12	Specify for each outcome the effect measure(s) (e.g. risk ratio, mean difference) used in the synthesis or presentation of results.	pp.4-5
Synthesis methods	13a	Describe the processes used to decide which studies were eligible for each synthesis (e.g. tabulating the study intervention characteristics and comparing against the planned groups for each synthesis (item #5)).	p.4
	13b	Describe any methods required to prepare the data for presentation or synthesis, such as handling of missing summary statistics, or data conversions.	pp.4-5
	13c	Describe any methods used to tabulate or visually display results of individual studies and syntheses.	p.4
	13d	Describe any methods used to synthesize results and provide a rationale for the choice(s). If meta-analysis was performed, describe the model(s), method(s) to identify the presence and extent of statistical heterogeneity, and software package(s) used.	pp.4-5
	13e	Describe any methods used to explore possible causes of heterogeneity among study results (e.g. subgroup analysis, meta-regression).	p.5

Section and Topic	Item #	Checklist item	Location where item is reported
	13f	Describe any sensitivity analyses conducted to assess robustness of the synthesized results.	p.5
Reporting bias assessment	14	Describe any methods used to assess risk of bias due to missing results in a synthesis (arising from reporting biases).	p.5
Certainty assessment	15	Describe any methods used to assess certainty (or confidence) in the body of evidence for an outcome.	p.5
**RESULTS**			
Study selection	16a	Describe the results of the search and selection process, from the number of records identified in the search to the number of studies included in the review, ideally using a flow diagram.	p.6
	16b	Cite studies that might appear to meet the inclusion criteria, but which were excluded, and explain why they were excluded.	p.6
Study characteristics	17	Cite each included study and present its characteristics.	pp.8-17
Risk of bias in studies	18	Present assessments of risk of bias for each included study.	p.24 and Table 2
Results of individual studies	19	For all outcomes, present, for each study: (a) summary statistics for each group (where appropriate) and (b) an effect estimate and its precision (e.g. confidence/credible interval), ideally using structured tables or plots.	Supp. Mat. Table S3
Results of syntheses	20a	For each synthesis, briefly summarise the characteristics and risk of bias among contributing studies.	Table 2 and 3
	20b	Present results of all statistical syntheses conducted. If meta-analysis was done, present for each the summary estimate and its precision (e.g. confidence/credible interval) and measures of statistical heterogeneity. If comparing groups, describe the direction of the effect.	Table 4.
	20c	Present results of all investigations of possible causes of heterogeneity among study results.	Table 4.
	20d	Present results of all sensitivity analyses conducted to assess the robustness of the synthesized results.	p. 21 and Table 4
Reporting biases	21	Present assessments of risk of bias due to missing results (arising from reporting biases) for each synthesis assessed.	p.21
Certainty of evidence	22	Present assessments of certainty (or confidence) in the body of evidence for each outcome assessed.	-
**DISCUSSION**			
Discussion	23a	Provide a general interpretation of the results in the context of other evidence.	pp.25-27
	23b	Discuss any limitations of the evidence included in the review.	p.26
	23c	Discuss any limitations of the review processes used.	p.26
	23d	Discuss implications of the results for practice, policy, and future research.	pp.26-27
**OTHER INFORMATION**			
Registration and protocol	24a	Provide registration information for the review, including register name and registration number, or state that the review was not registered.	Abstract and p.3
	24b	Indicate where the review protocol can be accessed, or state that a protocol was not prepared.	-
	24c	Describe and explain any amendments to information provided at registration or in the protocol.	p.4
Support	25	Describe sources of financial or non-financial support for the review, and the role of the funders or sponsors in the review.	Title page and (p.27)

Section and Topic	Item #	Checklist item	Location where item is reported
Competing interests	26	Declare any competing interests of review authors.	p.27
Availability of data, code and other materials	27	Report which of the following are publicly available and where they can be found: template data collection forms; data extracted from included studies; data used for all analyses; analytic code; any other materials used in the review.	p.27

From: Page MJ, McKenzie JE, Bossuyt PM, Boutron I, Hoffmann TC, Mulrow CD, et al. The PRISMA 2020 statement: an updated guideline for reporting systematic reviews. BMJ 2021;372:n71. doi: 10.1136/bmj.n71 [21]

Table S2 Literature Search Strategy

Database searched [date of search]	Search Terms	Filters / Limiters applied
PubMed [16.06.2022]	(toddler*[Title/Abstract] OR preschool[Title/Abstract] OR preschooler[Title/Abstract] OR "Child, Preschool"[MeSh Terms] OR "Infant"[mh:noexp]) AND ((("Infant Formula"[MeSH Terms] OR "Infant Food"[MeSH Terms] OR ("Milk"[MeSH Terms] AND "Food, Fortified"[MeSH Terms])) AND ("Vitamin D"[MeSH Terms] OR "Zinc"[MeSH Terms] OR "Ferrous Compounds"[MeSH Terms] OR "Phosphatidylcholines"[MeSH Terms] OR "Prebiotics"[MeSH Terms] OR "Probiotics"[MeSH Terms] OR "Synbiotics"[MeSH Terms])) OR ((milk[Title/Abstract] OR "growing up milk"[Title/Abstract] OR "growing-up milk"[Title/Abstract] OR "follow on formula"[Title/Abstract] OR "follow-on formula"[Title/Abstract]) AND (Formulated[Title/Abstract] OR formulation[Title/Abstract] OR supplemented[Title/Abstract] OR supplement[Title/Abstract] OR fortify[Title/Abstract] OR fortified[Title/Abstract] OR fortification[Title/Abstract] OR prebiotic*[Title/Abstract] OR probiotic*[Title/Abstract] OR synbiotic*[Title/Abstract]))) AND ("head circumference"[Title/Abstract] OR Growth [Title/Abstract] OR "Growth and Development"[MeSH Terms] OR "Growth"[MeSH Terms] OR "body size"[Title/Abstract] OR "Body Size"[MeSH Terms] OR weight[Title/Abstract] OR bodyweight[Title/Abstract] OR "Body Weight"[MeSH Terms] OR BMI[Title/Abstract] OR "body mass index"[Title/Abstract] OR "Body Mass Index"[MeSH Terms] OR "body composition"[Title/Abstract] OR "Body Composition"[MeSH Terms] OR "muscle mass"[Title/Abstract] OR "fat-free mass"[Title/Abstract] OR "fat free mass"[Title/Abstract] OR musculoskeletal[Title/Abstract] OR "Musculoskeletal Development"[MeSH Terms] OR "fat mass"[Title/Abstract] OR "fat free mass"[Title/Abstract] OR fat-free mass[Title/Abstract] OR "body fat"[Title/Abstract] OR "Adipose Tissue"[MeSH Terms] OR height[Title/Abstract] OR "Body Height"[MeSH Terms] OR length[Title/Abstract] OR Nutritional Status[MeSH Terms] OR "vitamin d"[Title/Abstract] OR zinc[Title/Abstract] OR iron[Title/Abstract] OR ferritin[Title/Abstract]) AND (randomized controlled trial[pt] OR controlled clinical trial[pt] OR clinical trial[pt] OR observational study[pt] OR randomi*[Title/Abstract] OR placebo*[Title/Abstract] OR clinical trials as topic[MeSH Terms:noexp] OR randomly[Title/Abstract] OR trial[Title/Abstract] OR trials[Title/Abstract] OR prospective[Title/Abstract] OR cross-over[Title/Abstract]) NOT ("animals"[MeSH Terms] NOT "humans"[MeSH Terms])	1990 onwards
Web of Science (Core Collection) [16.06.2022]	TS=(Toddler* OR Preschool OR Preschooler) AND TS=((milk OR "follow on formula" OR "follow-on formula" OR "fortified food") AND (formulated OR formulation OR supplemented OR supplement OR fortify OR fortified OR fortification OR prebiotic* OR probiotic* OR synbiotic*)) AND TS=("head circumference" OR growth OR "body size" OR weight OR bodyweight OR BMI OR "body mass index" OR "body composition" OR "muscle mass" OR "fat-free mass" OR "fat free mass" OR musculoskeletal OR "fat mass" OR "body fat" OR "adipose tissue" OR height OR length OR "nutritional status" OR "vitamin d" OR zinc OR iron OR ferritin) AND TS=("observational study" OR random* OR placebo* OR trial* OR prospective OR cross-over)	None
Scopus [16.06.2022]	(TITLE-ABS-KEY (toddler* OR preschool OR preschooler) AND TITLE-ABS-KEY ((milk OR "follow on formula" OR "follow-on formula") AND (formulated OR formulation OR supplemented OR supplement OR	None

	fortify OR fortified OR fortification OR prebiotic* OR probiotic* OR synbiotic*)) AND TITLE-ABS-KEY ("head circumference" OR growth OR "body size" OR weight OR bodyweight OR bmi OR "body mass index" OR "body composition" OR "muscle mass" OR "fat-free mass" OR "fat free mass" OR musculoskeletal OR "fat mass" OR "body fat" OR "adipose tissue" OR height OR length OR "nutritional status" OR "vitamin d" OR zinc OR iron OR ferritin) AND TITLE-ABS-KEY ("observational study" OR random* OR placebo* OR trial OR trials OR prospective OR cross-over) AND PUBYEAR > 1989)	
ProQuest (NTIS, Agriculture Science Database, Biological Science Database, Continental Europe Database, East & South Asia Database, East Europe, Central Europe Database, Health & Medical Collection, Healthcare Administration Database, India Database, Middle East & Africa Database, Nursing & Allied Health Database, Public Health Database, Science Database, Turkey Database, UK & Ireland Database) [16.06.2022]	(ti(Toddler* OR Preschool OR Preschooler) AND ti((milk OR "follow on formula" OR "follow-on formula") AND (formulated OR formulation OR supplemented OR supplement OR fortify OR fortified OR fortification OR prebiotic* OR probiotic* OR synbiotic*)) AND ti("head circumference" OR growth OR "body size" OR weight OR bodyweight OR BMI OR "body mass index" OR "body composition" OR "muscle mass" OR "fat-free mass" OR "fat free mass" OR musculoskeletal OR "fat mass" OR "body fat" OR "adipose tissue" OR height OR length "nutritional status" OR "vitamin d" OR zinc OR iron OR ferritin) AND ti("observational study" OR random* OR placebo* OR trial* OR prospective OR cross-over)) OR (ab(Toddler* OR Preschool OR Preschooler) AND ab((milk OR "follow on formula" OR "follow-on formula") AND (formulated OR formulation OR supplemented OR supplement OR fortify OR fortified OR fortification OR prebiotic* OR probiotic* OR synbiotic*)) AND ab("head circumference" OR growth OR "body size" OR weight OR bodyweight OR BMI OR "body mass index" OR "body composition" OR "muscle mass" OR "fat-free mass" OR "fat free mass" OR musculoskeletal OR "fat mass" OR "body fat" OR "adipose tissue" OR height OR length OR "nutritional status" OR "vitamin d" OR zinc OR iron OR ferritin) AND ab("observational study" OR random* OR placebo* OR trial* OR prospective OR cross-over))	1990 onwards
Google Scholar (via Publish or Perish) [16.06.2022]	(Toddler* OR Preschool OR Preschooler) AND ((milk OR "follow on formula" OR "follow-on formula" OR "fortified food") AND (formulated OR formulation OR supplemented OR supplement OR fortify OR fortified OR fortification OR prebiotic* OR probiotic* OR synbiotic*)) AND ("head circumference" OR growth OR "body size" OR weight OR bodyweight OR BMI OR "body mass index" OR "body composition" OR "muscle mass" OR "fat-free mass" OR "fat free mass" OR musculoskeletal OR "fat mass" OR "body fat" OR "adipose tissue" OR height OR length OR "nutritional status" OR "vitamin d" OR zinc OR iron OR ferritin) AND ("clinical trial*" OR "comparative stud*" OR "evaluation stud*" OR "controlled trial*" OR "follow-up stud*" OR "prospective stud*" OR random* OR placebo* OR "single blind* OR "double blind*" OR "observational study" OR cross-over OR crossover)	1990 onwards
Cochrane Library [16.06.2022]	Toddler* OR Preschool OR Preschooler in Title Abstract Keyword AND (milk OR "follow on formula" OR "follow-on formula" OR "fortified food") AND (formulated OR formulation OR supplemented OR supplement OR fortify OR fortified OR fortification OR prebiotic* OR probiotic* OR synbiotic*) in Title Abstract Keyword AND "head circumference" OR growth OR "body size" OR weight OR bodyweight OR BMI OR "body mass index" OR "body composition" OR "muscle mass" OR "fat-free mass" OR "fat free mass" OR musculoskeletal OR "fat mass" OR "body fat" OR "adipose tissue" OR height OR length OR "nutritional status" OR "vitamin d" OR zinc OR iron OR ferritin in Title Abstract Keyword AND	None

	"observational study" OR random* OR placebo* OR trial* OR prospective OR cross-over in Title Abstract Keyword - with Cochrane Library publication date Between Jan 1990 and Dec 2022 (Word variations have been searched)	
Clinical Trials [16.06.2022]	Toddler* OR Preschool OR Preschooler | ((milk OR "follow on formula" OR "follow-on formula") AND (formulated OR formulation OR supplemented OR supplement OR fortify OR fortified OR fortification OR prebiotic* OR probiotic* OR synbi*)) | head OR "body size" OR weight OR bodyweight OR BMI OR "body mass index" OR "body composition" OR "muscle mass" OR "fat-free" OR "fat free" OR musculoskeletal OR "fat mass" OR "body fat" OR height OR length OR nutrition* OR Zinc OR Iron OR "Vitamin d" | Child | First posted on or after 01/01/1990	Children only

Table S3 Summary of outcome measures reported across the articles and included in the review

Outcome measure	Articles reporting outcome (n, [references])	Articles included in the meta-analysis (n, [references])	Note
**Growth / Body Composition**			
Body weight	n=9 [1-9]	n=7 [1-7]	2 articles [8, 9] excluded due to duplicate data from another article (secondary analyses of [7] and [3], respectively)
Weight-for-age	n=7 [2, 4, 5, 7, 8, 10, 11]	n=4 [2, 4, 5, 7]	1 article [8] excluded due to duplicate data from another article (secondary analysis of [7])<br><br>1 article [10]^ excluded due to insufficient data reported<br><br>1 article [11]¢ excluded due to insufficient data reported
Length (/height)	n=7 [1-5, 7, 8, 10]	n=6 [1-5, 7]	1 article [8] excluded due to duplicate data from another article (secondary analysis of [7])
Length-(/height)-for-age	n=6 [4, 5, 7, 8, 10, 11]	n=3 [4, 5, 7]	1 article [8] excluded due to duplicate data from another article (secondary analysis of [7])<br><br>1 article [10]^ excluded due to insufficient data reported<br><br>1 article [11]¢ excluded due to insufficient data reported
Weight-for-length (/height)	n=6 [4, 5, 7, 8, 10, 11]	n=3 [4, 5, 7]	1 article [8] excluded due to duplicate data from another article (secondary analysis of [7])<br><br>1 article [10]^ excluded due to insufficient data reported<br><br>1 article [11]¢ excluded due to insufficient data reported
Body Mass Index	n=4 [1, 2, 7, 8]	n=3 [1, 2, 7]	1 article [8] excluded due to duplicate data from another article (secondary analysis of [7])
Body Mass Index-for-age	n=2 [7, 8]	N/A	1 article [8] excluded due to duplicate data from another article (secondary analysis of [7])

Head circumference	n=2 [2, 3]	N/A	Meta-analysis was not conducted on outcomes with <3 studies
Waist circumference, waist-height ratio	n=1 [7]	N/A	Meta-analysis was not conducted on outcomes with <3 studies
Mid-upper arm circumference	n=1 [3]	N/A	Meta-analysis was not conducted on outcomes with <3 studies
Skinfold thickness	n=1 [3]	N/A	Meta-analysis was not conducted on outcomes with <3 studies
Body fat %, fat mass, fat free mass, fat mass index	n=1 [8]	N/A	Meta-analysis was not conducted on outcomes with <3 studies
**Biochemical measures**			
Haemoglobin	n=11 [3-6, 9-15]	n=9 [3-6, 10, 12-15]	1 article [11][₵] excluded due to insufficient data reported<br><br>1 article [9] excluded due to duplicate data from another article (secondary analysis of [3])
Ferritin (serum)	n=11 [3-6, 9-15]	n=7 [4-6, 10, 12, 13, 15]	3 articles [3, 9, 14][¥] excluded due to insufficient data reported<br><br>1 article [11][₵] excluded due to insufficient data reported
Transferrin	n=4 [4-6, 13]	n=4 [4-6, 13]	-
Soluble transferrin receptors	n=6 [3, 6, 9, 11, 14, 15]	n=3 [6, 9, 15]	1 article [14][¥] excluded due to insufficient data reported<br><br>1 article [11][₵] excluded due to insufficient data reported<br><br>1 article [3][≠] excluded due to insufficient data reported
Vitamin D	n=3 [12, 13, 16]	N/A	1 article [16][¥] excluded due to insufficient data reported
Iodine (urinary)	n=1 [17]	N/A	Meta-analysis was not conducted on outcomes with <3 studies
Zinc	n=4 [4, 5, 10, 18]	N/A	1 article [18][ǂ] excluded due to insufficient data reported<br><br>2 articles [4, 5][§] excluded due to insufficient data reported

**Dietary intake**			
Energy	n=6 [4, 8, 9, 17-19]	N/A	1 article [18] excluded due to duplicate data from another article [17]<br><br>1 article [8] excluded due to duplicate data from another article [19]<br><br>Data provided by the other four studies [4, 9, 17, 19] are unable to be meta-analysed due to incorrect format[≠]
Macronutrients	Protein, n=3 [4, 8, 19]	N/A	1 article [8] excluded due to duplicate data from another article [19]
	Fat, n=2 [4, 19]	N/A	
	Carbohydrate, n=2 [4, 19]	N/A	
Micronutrients	Iron, n=5 [4, 6, 13, 14, 19]	N/A	1 article [13] excluded due to duplicate data from another article [19]<br><br>1 article [14] [*] excluded due to insufficient data reported<br><br>Data provided by the other three studies[4, 6, 19] are unable to be meta-analysed due to incorrect format[≠]
	Zinc, n=2 [4, 19]	N/A	
	Calcium, n=1 [19]	N/A	
	Vit B_{12}, n=1 [19]	N/A	
	Vit C, n=1 [19]	N/A	
	Vit D, n=1 [19]	N/A	

Note: ǂ, data reported as percentage of change, calculated as log-transformed values of intervention group minus that of control group, and expressed as percentage; §, article did not report a measure of variability (e.g., SD or 95% CI) for the group change and could not be imputed from other studies; ^, data reported by gender not whole group; ¥, data reported as geometric mean; ¢, data not shown; ≠, missing data (baseline or post values missing); *, data cannot accurately be extrapolated from figure.

Abbreviations: N/A, not applicable.

First author, year	Domain 1	Domain 2	Domain 3	Domain 4	Domain 5	Overall
Akkermans et al., 2017	!	+	+	+	+	!
Chatchatee et al., 2014	+	+	+	+	!	!
Firmansyah et al., 2011	+	+	+	+	!	!
Pontes et al., 2016	+	+	+	+	!	!
Rivera et al., 2010	+	+	+	+	!	!
Sazawal et al., 2010a	+	+	+	+	!	!
Sazawal et al., 2010b	+	+	+	+	+	+
Villalpando et al., 2006	!	+	+	!	-	-
Virtanen et al., 2001	+	+	-	+	!	-
GUMLi trial						
Wall et al., 2019	+	+	+	+	!	!
Lovell et al., 2018	+	+	+	+	!	!
Lovell et al., 2019	+	+	+	+	!	!
Lovell et al., 2021	+	+	+	+	!	!
Large UK trial						
Morley et al., 1999	+	+	+	+	+	+
Singhal et al., 2000	+	+	+	+	+	+
Toddler Food Study						
Szymlek-Gay et al., 2009	+	+	+	+	+	+
Morgan et al., 2010	+	+	+	+	!	!
Houghton et al., 2011	+	+	+	+	!	!
Szymlek-Gay et al., 2020	+	+	+	+	!	!

Legend

+ Low risk
! Some concerns
- High risk

Figure S1 Quality assessment of the included studies using the Cochrane Risk of Bias 2.0 Tool

Note: Domain 1, Randomisation process; Domain 2, Deviations from the intended interventions; Domain 3, Missing outcome data; Domain 4, Measurement of the outcome; Domain 5, Selection of the reported result

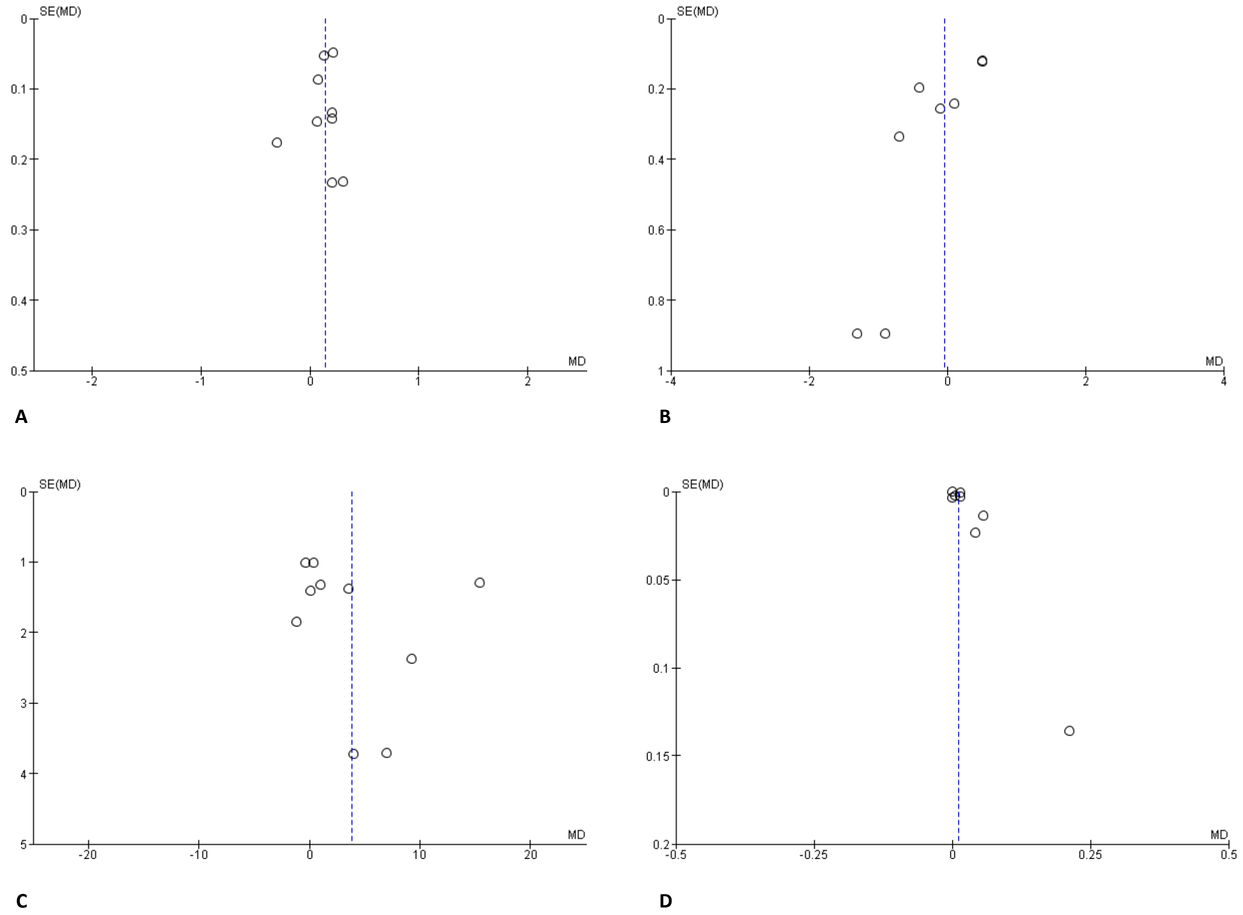

Figure S2 Funnel plots of intervention effect estimates (mean difference) from individual studies against the standard error of the effect estimate. Mean differences (x-axis) for (A) body weight (kg); (B) length (cm); (C) haemoglobin; (D) ferritin are plotted against the standard error (y-axis).

Note: Scatter dots represent individual studies, dashed diagonal lines indicates pseudo 95% Confidence Interval. Funnel plot presented for outcomes with ≥5 comparisons.

Abbreviations: SE, Standard Error; MD, Mean Difference.

References

1. Chatchatee P, Lee WS, Carrilho E, Kosuwon P, Simakachorn N, Yavuz Y, et al. Effects of growing-up milk supplemented with prebiotics and LCPUFAs on infections in young children. J Pediatr Gastroenterol Nutr. 2014;58(4):428-37.
2. Firmansyah A, Dwipoerwantoro PG, Kadim M, Alatas S, Conus N, Lestarina L, et al. Improved growth of toddlers fed a milk containing synbiotics. Asia Pac J Clin Nutr. 2011;20(1):69-76.
3. Morley R, Abbott R, Fairweather-Tait S, MacFadyen U, Stephenson T, Lucas A. Iron fortified follow on formula from 9 to 18 months improves iron status but not development or growth: a randomised trial. Arch Dis Child. 1999;81(3):247-52.
4. Sazawal S, Dhingra U, Dhingra P, Hiremath G, Sarkar A, Dutta A, et al. Micronutrient fortified milk improves iron status, anemia and growth among children 1-4 years: a double masked, randomized, controlled trial. PLoS One. 2010;5(8):e12167.
5. Sazawal S, Dhingra U, Hiremath G, Sarkar A, Dhingra P, Dutta A, et al. Effects of Bifidobacterium lactis HN019 and prebiotic oligosaccharide added to milk on iron status, anemia, and growth among children 1 to 4 years old. J Pediatr Gastroenterol Nutr. 2010;51(3):341-6.
6. Virtanen MA, Svahn CJ, Viinikka LU, Räihä NC, Siimes MA, Axelsson IE. Iron-fortified and unfortified cow's milk: effects on iron intakes and iron status in young children. Acta Paediatr. 2001;90(7):724-31.
7. Wall CR, Hill RJ, Lovell AL, Matsuyama M, Milne T, Grant CC, et al. A multicenter, double-blind, randomized, placebo-controlled trial to evaluate the effect of consuming Growing Up Milk "Lite" on body composition in children aged 12-23 mo. Am J Clin Nutr. 2019;109(3):576-85.
8. Lovell AL, Milne T, Matsuyama M, Hill RJ, Davies PSW, Grant CC, et al. Protein Intake, IGF-1 Concentrations, and Growth in the Second Year of Life in Children Receiving Growing Up Milk - Lite (GUMLi) or Cow's Milk (CM) Intervention. Front Nutr. 2021;8:666228.
9. Singhal A, Morley R, Abbott R, Fairweather-Tait S, Stephenson T, Lucas A. Clinical safety of iron-fortified formulas. Pediatrics. 2000;105(3):E38.
10. Pontes MV, Ribeiro TC, Ribeiro H, de Mattos AP, Almeida IR, Leal VM, et al. Cow's milk-based beverage consumption in 1- to 4-year-olds and allergic manifestations: an RCT. Nutr J. 2016;15:19.
11. Rivera JA, Shamah T, Villalpando S, Monterrubio E. Effectiveness of a large-scale iron-fortified milk distribution program on anemia and iron deficiency in low-income young children in Mexico. Am J Clin Nutr. 2010;91(2):431-9.
12. Akkermans MD, Eussen SR, van der Horst-Graat JM, van Elburg RM, van Goudoever JB, Brus F. A micronutrient-fortified young-child formula improves the iron and vitamin D status of healthy young European children: a randomized, double-blind controlled trial. Am J Clin Nutr. 2017;105(2):391-9.
13. Lovell AL, Davies PSW, Hill RJ, Milne T, Matsuyama M, Jiang Y, et al. Compared with Cow Milk, a Growing-Up Milk Increases Vitamin D and Iron Status in Healthy Children at 2 Years of Age: The Growing-Up Milk-Lite (GUMLi) Randomized Controlled Trial. J Nutr. 2018;148(10):1570-9.
14. Szymlek-Gay EA, Ferguson EL, Heath ALM, Gray AR, Gibson RS. Food-based strategies improve iron status in toddlers: a randomized controlled trial. American Journal of Clinical Nutrition. 2009;90(6):1541-51.
15. Villalpando S, Shamah T, Rivera JA, Lara Y, Monterrubio E. Fortifying milk with ferrous gluconate and zinc oxide in a public nutrition program reduced the prevalence of anemia in toddlers. J Nutr. 2006;136(10):2633-7.
16. Houghton LA, Gray AR, Szymlek-Gay EA, Heath AL, Ferguson EL. Vitamin D-fortified milk achieves the targeted serum 25-hydroxyvitamin D concentration without affecting that of parathyroid hormone in New Zealand toddlers. J Nutr. 2011;141(10):1840-6.
17. Szymlek-Gay EA, Gray AR, Heath AM, Ferguson EL, Edwards T, Skeaff SA. Iodine-fortified toddler milk improves dietary iodine intakes and iodine status in toddlers: a randomised controlled trial. Eur J Nutr. 2020;59(3):909-19.
18. Morgan EJ, Heath AL, Szymlek-Gay EA, Gibson RS, Gray AR, Bailey KB, et al. Red meat and a fortified manufactured toddler milk drink increase dietary zinc intakes without affecting zinc status of New Zealand toddlers. J Nutr. 2010;140(12):2221-6.
19. Lovell AL, Davies PSW, Hill RJ, Milne T, Matsuyama M, Jiang Y, et al. A comparison of the effect of a Growing Up Milk-Lite (GUMLi) v. cows' milk on longitudinal dietary patterns and nutrient intakes in children aged 12-23 months: The GUMLi randomised controlled trial. British Journal of Nutrition. 2019;121(6):678-87.
